# Photocrosslinkable Gelatin Hydrogels Modulate the Production of the Major Pro-inflammatory Cytokine, TNF-α, by Human Mononuclear Cells

**DOI:** 10.3389/fbioe.2018.00116

**Published:** 2018-09-19

**Authors:** Amy R. Donaldson, Constantin Edi Tanase, Dennis Awuah, Pranav Vasanthi Bathrinarayanan, Laurence Hall, Mehdi Nikkhah, Ali Khademhosseini, Felicity Rose, Cameron Alexander, Amir M. Ghaemmaghami

**Affiliations:** ^1^Immunology and Tissue Modelling Group, School of Life Sciences, University of Nottingham, Nottingham, United Kingdom; ^2^School of Biological and Health Systems Engineering, Arizona State University, Tempe, AZ, United States; ^3^Center for Minimally Invasive Therapeutics (C-MIT), California NanoSystems Institute (CNSI), University of California, Los Angeles, Los Angeles, CA, United States; ^4^Division of Regenerative Medicine and Cellular Therapies, School of Pharmacy, University of Nottingham, Nottingham, United Kingdom; ^5^Division of Molecular Therapeutics and Formulation, School of Pharmacy, University of Nottingham, Nottingham, United Kingdom

**Keywords:** tissue engineering, biomaterials, immunology, Gelatin methacryloyl, TNF-α

## Abstract

Hydrogels are an attractive class of biomaterials in tissue engineering due to their inherently compatible properties for cell culture. Gelatin methacryloyl (GelMA) has shown significant promise in the fields of tissue engineering and drug delivery, as its physical properties can be precisely tuned depending on the specific application. There is a growing appreciation for the interaction between biomaterials and cells of the immune system with the increasing usage of biomaterials for *in vivo* applications. Here, we addressed the current lack of information regarding the immune-modulatory properties of photocrosslinked GelMA. We investigated the ability of human mononuclear cells to mount inflammatory responses in the context of a GelMA hydrogel platform. Using lipopolysaccharide to stimulate a pro-inflammatory immune response, we found tumor necrosis factor-α (TNF-α) expression was suppressed in GelMA culture conditions. Our findings have important implications on the future use of GelMA, and potentially similar hydrogels, and highlight the significance of investigating the potential immune-modulatory properties of biomaterials.

## Introduction

Hydrogels are widely used in the field of tissue engineering and regenerative medicine (El-Sherbiny and Yacoub, 2013). They are an attractive class of biomaterials since their highly hydrated structure closely resembles the natural extracellular matrix (ECM) of soft tissues (Slaughter et al., 2009). A diverse range of hydrogels, formulated from natural and synthetic polymers, has been developed for tissue engineering applications (Annabi et al., 2014).

Gelatin methacryloyl (GelMA) is a hydrogel which has shown promise in various areas of tissue engineering including cardiac (Shin et al., 2013; Saini et al., 2015), bone (Ovsianikov et al., 2011; Dolatshahi-Pirouz et al., 2014), and vascularization (Chen et al., 2012; Bertassoni et al., 2014) as well as development of tumor microenvironment models (Peela et al., 2016). Derived from collagen, a naturally abundant ECM protein, GelMA has inherently biocompatible properties (Hutson et al., 2011). This includes the preservation of cell adhesion motifs, these specific sequences of amino acids are recognized by various cell-surface ECM receptors, such as integrins (Knight et al., 2000), which mediate cell attachment, motility, survival, and differentiation (Rosso et al., 2004).

Crosslinked GelMA has been used successfully in a variety of tissue engineering applications. For instance, Nikkhah et al. demonstrated the application of micropatterned GelMA in an investigation into the impact of geometry on endothelial cells in the context of vasculature organization and development (Nikkhah et al., 2012). Also, due to its mechanical stability, GelMA is an attractive material for incorporation into microfluidic systems. This has been successfully implemented by Chen et al. in the development of a microfluidic device which models the heart valve microenvironment (Chen et al., 2013). Furthermore, the *in vitro cell-compatibility* of GelMA has been established with a range of cell types including NIH 3T3 fibroblasts (Aubin et al., 2010), immortalized human umbilical vein endothelial cells (Nichol et al., 2010), monocytic cell line THP-1 (Cha et al., 2017), mesenchymal stem cells (Chen et al., 2012), and myoblasts (Ramón-Azcón et al., 2012).

The synthesis of GelMA involves chemical modification of gelatin by methacryloyl derivatives resulting in the formation of polymerisable methacrylamide or methacrylate side groups. These side-chains enable tuning of the physical properties of GelMA derivatives in a way not possible over its unmodified counterparts, collagen and gelatin. Thus, the mechanical strength and degradability of GelMA hydrogels may be controlled depending on the desired application (Van Den Bulcke et al., 2000). Although methacryloyl-functionalised gelatin retains properties similar to unmodified gelatin, polymerisation gives rise to a highly crosslinked, stiffer hydrogel (Van Den Bulcke et al., 2000). For example, with the addition of a photoinitiator, GelMA is photo-polymerisable upon exposure to an ultra violet (UV) light source. Thus, soft lithography micropatterning techniques can be used to achieve a high level of control over the architectural features of GelMA hydrogels (Nichol et al., 2010).

GelMA is evidently compatible with a range of cell types, however, there is a current lack of information regarding its interactions with immune cells. The requirement of understanding how materials of this type interact with the immune system is largely being driven by the increasing usage of synthetic materials *in vivo*, as vehicles for cell, protein and DNA delivery.

Immune cells have a natural propensity to mount responses against foreign bodies, which extends to biomaterials. Briefly, upon recognition of a foreign material, cells of the immune system (e.g., antigen presenting cells such as monocytes and macrophages), become activated in order to attempt to eradicate it by phagocytosis (Anderson et al., 2008; Ekdahl et al., 2011). Production of cytokines including tumor necrosis factor (TNF)-α, interleukin (IL)-1β, IL-6, and IL-8 (Gretzer et al., 2003), by activated cells are characteristic of this response and mediate inflammation and wound healing *in vivo* (Chang et al., 2008).

Recently, certain biomaterials, such as poly(lactic-co-glycolic acid) based particles, have been shown to have profound effects in terms of modulating the immune system (Yoshida and Babensee, 2004; Silva et al., 2015). These properties can be harnessed in areas such as vaccine delivery, autoimmune disease and cancer therapeutics to direct appropriate immune responses *in vivo*. In the development of vaccines, for example, biomaterial adjuvanticity enhances the induction of adaptive immune responses, thereby potentially increasing the level of protection offered by the vaccine (Lewis et al., 2014).

To the best of our knowledge, despite its wide usage in the field of tissue engineering, the immunological properties of GelMA have not yet been characterized. We addressed this by assessing the interaction between GelMA hydrogels and human primary peripheral blood mononuclear cells (PBMCs) with a focus on GelMA's ability to induce and/or modulate immune responses. To do this, we measured the elicitation of an immune reaction to GelMA based on the production of pro-inflammatory cytokines. In addition, to investigate whether GelMA affects the ability of immune cells to mount appropriate immune responses, we used lipopolysaccharide (LPS) to elicit an inflammatory reaction (Donaldson, 2017). We anticipate the findings from this study will have important implications on the future applications of GelMA and potentially similar hydrogels in tissue engineering.

## Materials and methods

### PBMC preparation and monocyte isolation

Heparinized blood from healthy donors was obtained with consent and ethical committee approval. PBMCs were separated on a Histopaque-1077 (Sigma-Aldrich) density gradient as described before (García-Nieto et al., 2010). In some experiments we used CD14^+^ monocytes that were isolated using magnetic assisted cell sorting (MACS) and CD14 conjugated magnetic beads (Miltenyi Biotec, UK) as we have previously described (Al-Ghouleh et al., 2012; Salazar et al., 2017).

### Methacryloyl gelatin (GelMA) hydrogel preparation

The GelMA foam was synthesized as described previously (Van Den Bulcke et al., 2000) and kindly provided by the Khademhosseini Lab. The photoinitiator, 2-Hydroxy-4′-(2-hydroxyethoxy)-2-methylpropiophenone (Irgacure 2959) (Sigma-Aldrich, UK) was dissolved in PBS to make a 0.25% (w/v) solution. A 5% (w/v) GelMA pre-polymer was prepared by dissolving GelMA into the 0.25% photoinitiator solution at 60°C. One hundred microliter of pre-polymer solution was added per well of a 48-well tissue culture plate and photo-cross linked by UV exposure (40 s, 800 mW, 8 cm). Hydrogels were washed in PBS and sterilized by submersion in a 20% antibiotic\antimycotic solution overnight. A final wash was done in PBS before cell seeding.

### Cell culture on hydrogels

PBMCs or purified monocytes (5 × 10^5^) were seeded per gel in 500 μL RPMI-1640 supplemented with 100 U/mL penicillin, 100 mg/mL streptomycin, 2 mM L-glutamine, and 10% fetal calf serum (FCS) (all purchased from Sigma Aldrich). For the simulated conditions, 0.1 μg/mL *E. coli* LPS (0111:B4) was added to the appropriate wells. Plates were transferred to a humidified incubator (37°C, 5% CO_2_). Cultures were maintained for up to 5 days to assess cell viability. Supernatant sampled after 4 and 24 h for cytokine analysis.

CD14^+^ monocytes were stimulated with LPS for 1hr in polypropylene tubes prior to culture on TC, GelMA or gelatin coated well plates for 4 and 24h (*n* = 6). Since the CD14^+^ monocytes are stimulated with LPS prior to transfer to different substrates the level of LPS stimulation is considered to be similar for all conditions (GelMA, Gelatin, and TC). One way ANOVA- Tukey's multiple comparisons test was used to assess statistical significance (^****^P ≤ 0.0001).

### Cell viability assay

To assess the viability of cells on day 5 of culture on hydrogel substrates the Annexin V-FITC cell viability kit (purchased from Beckman Coulter) was used. This assay is based on the principle that in early apoptosis cells lose the asymmetry of the plasma membrane. Thus, phosphotidylserine (PS), normally located on the inside of the cell membrane, appears on the outer surface. Annexin V has a high affinity for PS, therefore when conjugated with a fluorochrome (e.g., FITC), can be used to label cells in early apoptosis. During late apoptosis, the integrity of the cell membrane is lost allowing penetration of propidium iodide (PI), which intercalates DNA. Upon DNA binding, PI fluoresces allowing detection of non-viable cells. Cells were harvested from the gels by gentle washing with ice cold PBS and immediately placed on ice. After washing, cells were re-suspended in 1X binding buffer and the staining method was carried out according to manufacturer's instructions. Samples were analyzed on the Beckman Coulter FC500.

### Live/dead assay

Cells grown on GelMA and tissue culture plastic were subjected to a LIVE/DEAD assay (L3224) according to manufacturer's guidelines (Thermo Fisher). Cells were imaged using a ZOE Fluorescent Cell Imager microscope (Bio-Rad, UK).

### TNF-α depletion experiments

Recombinant human TNF-α (purchased from Invitrogen) was added to complete RPMI-1640 media at either 5 or 2.5 ng/mL. GelMA hydrogels were made as described above in the GelMA hydrogel preparation section. Five hundred microliter of media with or without the addition of TNF-α was incubated with the gels for 4 h in a humidified incubator (37°C, 5% CO_2_). Supernatant was collected and stored at −80°C for cytokine analysis.

### Cytokine analysis

Interleukin 1β, interleukin 6, interleukin 8 and tumor necrosis factor-α were measured using a multiplex bead-based analyte detection system according to manufacturer's instructions (Procartaplex) (purchased from eBioscience) as we have described before (Sharquie et al., 2013). Supernatants from cell-free TNF-α depletion experiments were analyzed for TNF-α by ELISA following manufacturer's instructions (Human TNF-α DuoSet) (purchased from R&D systems).

### Preparation of thin hydrogel layers for immuno-fluorescent staining

GelMA was photo-polymerised on surface-treated glass chips cut to 1 cm^2^. Glass slides were coated with 3-(Trimethoxysilyl) propyl methacrylate (TMSPMA) in order to functionalise the surface to enhance hydrogel attachment. Briefly, glass microscope slides were submerged in a 10% (w/v) sodium hydroxide solution overnight. Slides were thoroughly rinsed and soaked overnight in diH_2_O. They were washed 3 times in 100% ethanol and air-dried. Slides were then stacked in a beaker and wetted with TMSPMA. The beaker was covered with aluminum foil and the slides were baked at 80°C overnight. Finally, slides were washed in 100% ethanol 3 times, wrapped in aluminum foil and baked for a further 2 h at 80°C. A 5% (w/v) pre-polymer GelMA solution was prepared as described previously. Five microliter gel solution was dispensed onto a glass chip, a spacer with a depth of 100 μm was placed on top to form an even layer and then the gel was crosslinked for 8.5 s (Nikkhah et al., 2012). The glass chips were transferred to a 24 well plate and washed with PBS. Then gels were submerged in control media or media containing recombinant human TNF-α and incubated for 4 h in a humidified incubator (37°C, 5% CO_2_).

### Immuno-fluorescent staining of GelMA-bound TNF-α

Following a 4 h incubation with or without TNF-α-containing media, the supernatant was removed and the gels were washed in PBS. The samples were blocked for 30 min with 5% (w/v) FCS in PBS. A 1:200 dilution [in 5% (w/v) FCS in PBS] was made for the mouse anti-human TNF-α monoclonal antibody (purchased from abcam) and incubated with samples overnight at 4°C. Samples were washed 3 times in PBS and then incubated for 1 h with a Rhodamine red-conjugated goat anti-mouse secondary antibody (purchased from Life Technologies) diluted 1:250 with 5% (w/v) FCS in PBS. Finally, samples were washed in PBS and mounted for imaging using a 20x objective on a Zeiss LSM710 confocal microscope.

### mRNA isolation and cDNA synthesis

Cells were harvested from hydrogel substrates by washing with cold PBS and immediately transferred to a Falcon tube on ice. Cells were washed twice in PBS, then 1 mL of MACS lysis/ re suspension buffer was added to 10^7^ cells and mixed until lysate was clear. mRNA purification and cDNA synthesis were carried out using the μMACS one-step cDNA kit (Miltenyi Biotech, UK) following the manufacturer‘s instructions. The purity and quantity of the cDNA was assessed with a NanoDrop 1000 Spectrophotometer (Thermo Scientific).

### Conventional PCR

Conventional PCR was carried out in a TC-312 PCR Thermocycler (Bibby Scientific Ltd., UK) using the Phusion Flash High-Fidelity PCR Master Mix (Thermo Fisher Scientific) and 20 ng of μMACS cDNA per reaction. Primers were obtained from Eurofin, UK (Table 1): PCR was carried out with an initial denaturation at 98°C for 10 s, followed by 32 cycles of denaturation (98°C, 0–1 s), annealing (62°C, 30 s), extension (72°C, 30 s), final extension was performed at 72°C for 60 s. Then, the PCR products were analyzed in an E-gel pre-cast 2% agarose electrophoresis system (Thermo Fisher Scientific) and the molecular weight of the bands were calculated with a standard 100 bp Directload DNA ladder (Sigma-Aldrich).

**Table 1:** Primers for real-time PCR.

**Genes**	**Primer**	**Sequence (5^′^- 3^′^)**
GAPDH	Forward	GAGTCAACGGATTTGGTCGT
	Reverse	GACAAGCTTCCCGTTCTCAG
TNF-α	Forward	CAGAGGGAAGAGTTCCCCAG
	Reverse	CCTTGGTCTGGTAGGAGACG

### Quantitative real-time PCR analysis

Real time PCR was performed in a Strategene MxPro 3005P qPCR System with the Brilliant III Ultra-Fast SYBR Green qPCR Master Mix (Agilent Technologies, USA). Primers were obtained from Eurofin, details for GAPDH and TNF-α as above. Cycling was initiated at 95°C for 3 min, followed by 45 cycles of 95°C for 20 s and 62°C for 30 s, a melting curve was done at the end. Samples were run in triplicates and relative expression calculated using comparative threshold cycle method normalized to GAPDH (Breloer and Fleischer, 2008; Benton et al., 2009). For all the experiments, three independent donors were used.

### Statistical analysis

Analysis was carried out using GraphPad Prism version 6.00 for Windows, GraphPad Software, La Jolla California USA (www.graphpad.com). Results are expressed as mean values ± standard deviation (SD) from three or more independent experiments. Statistical differences were determined using the student *t* test or one-way ANOVA with Tukey *post-hoc* testing. A *p*-value <0.05 was considered statistically significant. Data shown is indicative of three independent experiments with three donors, unless stated otherwise.

## Results

### GelMA hydrogels do not have a detrimental impact on immune cell viability

**Figure 1:**
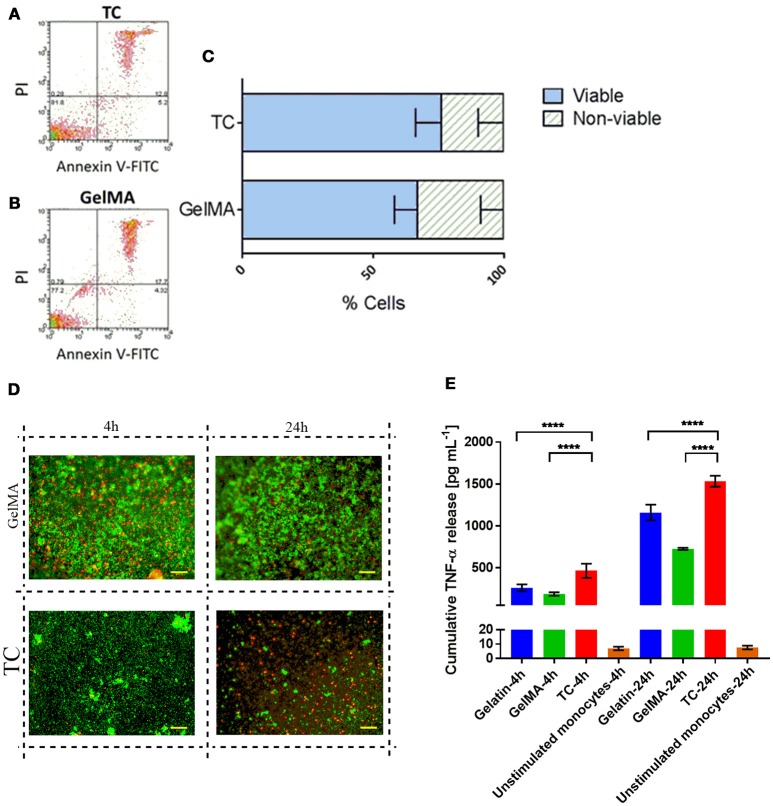
PBMC viability is not impaired by culture with GelMA hydrogels. **(A,B)** Annexin V-FITC/PI staining profiles of cells harvested from TC and GelMA cultures respectively. **(C)** Percentage of viable (dark gray bars) and non-viable (light gray bars) cells collected from GelMA and TC. Two-tailed *t*-tests determined that there were no significant differences (*P* > 0.05) in the proportion of viable cells, or dead cells harvested from GelMA cultures compared to TC. Error bars represent standard deviation (*n* = 3). **(D)** Live/Dead staining of PBMC on GelMA and TC for 4 and 24 h (scale bar 100 μm) where green fluorescent highlights live and red highlights dead cells respectively. **(E)** Measurement of TNF-α production by LPS stimulated CD14^+^ monocytes seeded on different substrates. ****p < 0.0001.

PBMCs, isolated from the blood of healthy donors, were used as a source of primary human immune cells in our experiments since it comprises the cell types of interest for this work. The cellular composition of PBMCs is 70–90% lymphocytes [T cells, B cells, and natural killer (NK) cells], 3–10% monocytes, 1–2% dendritic cells, and small numbers of basophils. Figure 1 shows the results from the cytotoxicity and viability assays on PBMCs [cultured on tissue culture plastic (TC) or GelMA] using Annexin V-FITC/PI staining (Figures 1A–C) and LIVE/DEAD staining respectively (Figure 1D). Data from both assays indicate comparable levels of viability in cells cultured on TC or GelMA. Data with CD14^+^ monocytes shows that detectable soluble TNF-α for cells transferred to GelMA is significantly lower than cells transferred to TC (*p* < 0.0001) for both time points. This suggests that GelMA potentially “mops up” the TNF-α released in the medium by CD14^+^ monocytes. Interestingly enzymatically cross-linked gelatin hydrogels seem to have a similar effect on soluble TNF-α (Paguirigan and Beebe, 2007).

### Characterizing the immune-modulatory properties of GelMA

**Figure 2:**
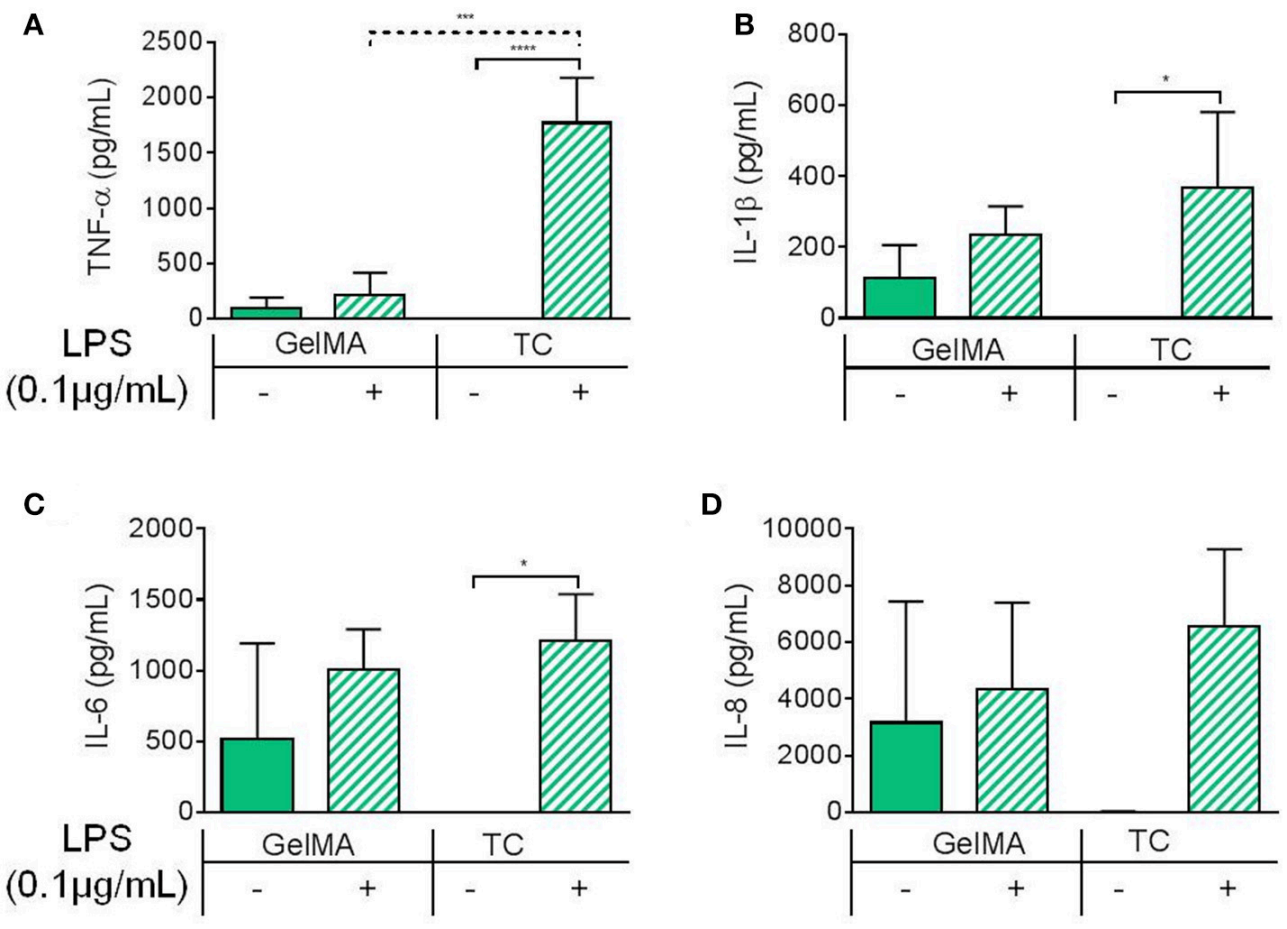
Inflammatory cytokine production by PBMCs cultured on GelMA hydrogels. Soluble cytokine production after 4 h by PBMCs cultured on GelMA hydrogels or TC, with (+) or without (–) LPS stimulation. **(A)** TNF-α was not significantly increased following LPS stimulation in GelMA cultures. Secretion of TNF-α, into the cell culture medium, was found to increase significantly in the LPS-stimulated TC control (****P ≤ 0.0001). A significant difference in the level of LPS-induced TNF-α production was found between GelMA and TC conditions (***P ≤ 0.001). **(B)** Production of IL-1β was not significantly increased with LPS stimulation in the presence of GelMA, whereas in TC it was (* ≤ 0.05). **(C)** IL-6 secretion was not significantly increased in GelMA cultures following LPS stimulation, this was found to be significant in TC (*P ≤ 0.05). **(D)** No significant rise in IL-8 production with LPS stimulation GelMA or TC cultures (*P* ≥ 0.05). One-way ANOVA performed to determine statistically significant differences in cytokine production (*n* = 3 independent experiments). Error bars represent standard deviation. **p < 0.01.

To characterize the immune-modulatory properties of GelMA, we investigated the responsiveness of PBMCs to immunological manipulation in the presence of GelMA (Figure 2). This was done by assessing the response of PBMCs to lipopolysaccharide (LPS), an immunogenic agent derived from the outer membrane of Gram-negative bacteria. PBMCs were cultured on TC or GelMA hydrogels, with or without LPS for 4 h. Secretion of a panel of inflammatory cytokines (IL-1β, IL-6, IL-8, and TNF-α) was used to assess the immune response (Figures 2A–D). In cells cultured on TC, levels of all cytokines increased significantly in response to LPS stimulation. In the presence of GelMA hydrogels, background levels of all inflammatory cytokines were raised. Production of IL1-β, IL-6, and IL-8 increased to some extent with LPS stimulation, although the differences were not statistically significant. Crucially, there was no change in the level of TNF-α detected following stimulation by LPS.

To assess whether the main responders to LPS stimulation in these experiments are monocytes and to rule out low LPS availability as the reason for low TNF-α levels in cells stimulated on GelMA, we repeated these experiments using purified monocytes that were stimulated with LPS prior to transfer to TC plates or GelMA coated wells. These data showed the same pattern of TNF-α production with significantly lower levels of soluble TNF-a detected in the supernatant of cells transferred to GelMA coated plates compared to TC (Figure 1E).

### GelMA suppresses LPS-induced TNF-α gene expression in PBMCs

**Figure 3:**
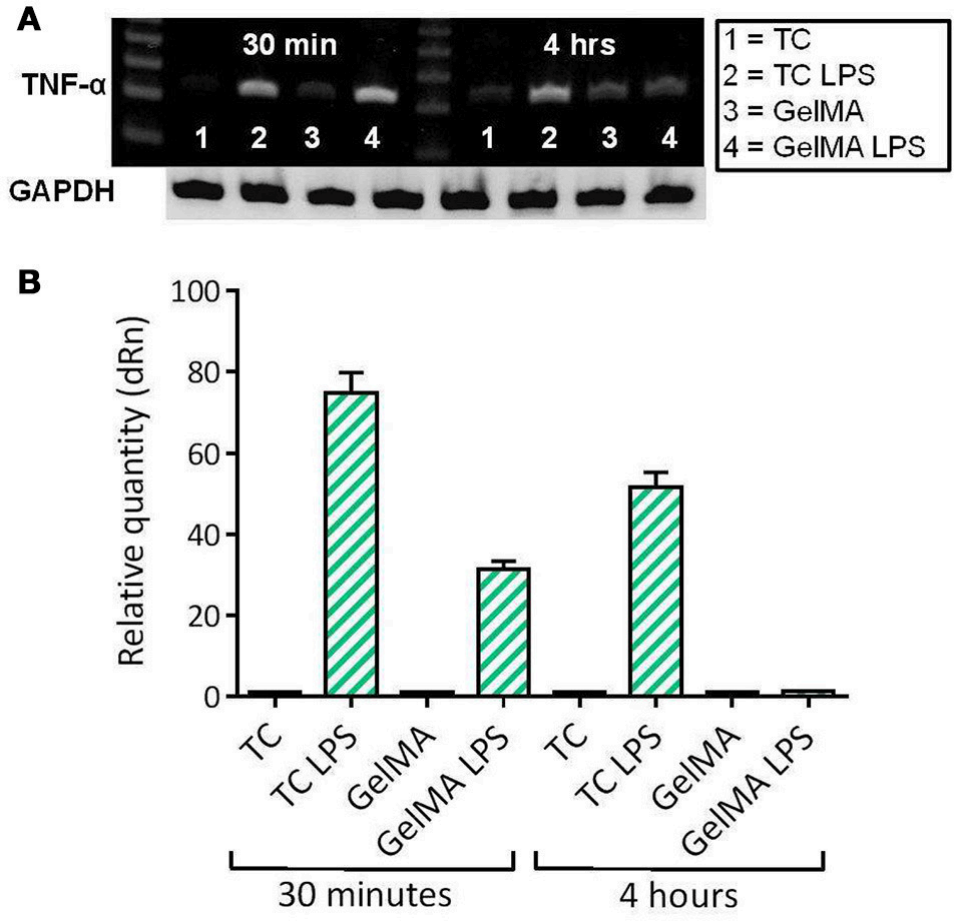
LPS-induced TNF-α gene expression is down-regulated in PBMCs cultured on GelMA. **(A)** Measurement of TNF-α gene expression by conventional ethidium bromide gel-based RT PCR. After 30 min in culture, the band intensity indicates TNF-α gene expression has increased as a result of LPS stimulation in both GelMA and TC cultures. At 4 h, expression of TNF-α was maintained in the TC control, however, it was down-regulated in the GelMA condition. **(B)** Analysis of TNF-α gene expression by real time quantitative PCR. Thirty minute after LPS stimulation the relative quantity of TNF-α expression increased in GelMA and TC cultures. After 4 h, up-regulation of TNF-α gene expression was maintained in TC cultures, however, LPS-induced TNF-α gene expression was attenuated in the presence of GelMA. ΔΔ Ct Analysis normalized against GAPDH. Data shown is representative of 3 independent donors.

Given the observation that GelMA has potentially suppressive effects on levels of LPS-induced TNF-α, further investigation at the level of gene expression was carried out. In these experiments, PBMCs were cultured on photo-polymerised GelMA hydrogel layers, with and without LPS. The cells were then harvested for analysis of TNF-α gene expression after 30 min and 4 h of culture. TNF-α gene expression by PBMCs in culture with GelMA hydrogels was then measured by both conventional gel-based and reverse transcription PCR (Figure 3). As shown, TNF-α expression is substantially induced at 30 min following LPS stimulation in both TC and GelMA; however, after 4 h TNF-α mRNA expression in cells cultured on GelMA is reduced to the levels observed in un-stimulated cells (Figure 3B).

### GelMA hydrogels “mop up” TNF-α

We used a cell-free system to determine whether soluble TNF-α protein is depleted by GelMA. In these experiments, recombinant human TNF-α was added to the tissue culture media and incubated with GelMA hydrogels. After 4 h, the supernatants were collected for cytokine analysis by ELISA.

**Figure 4:**
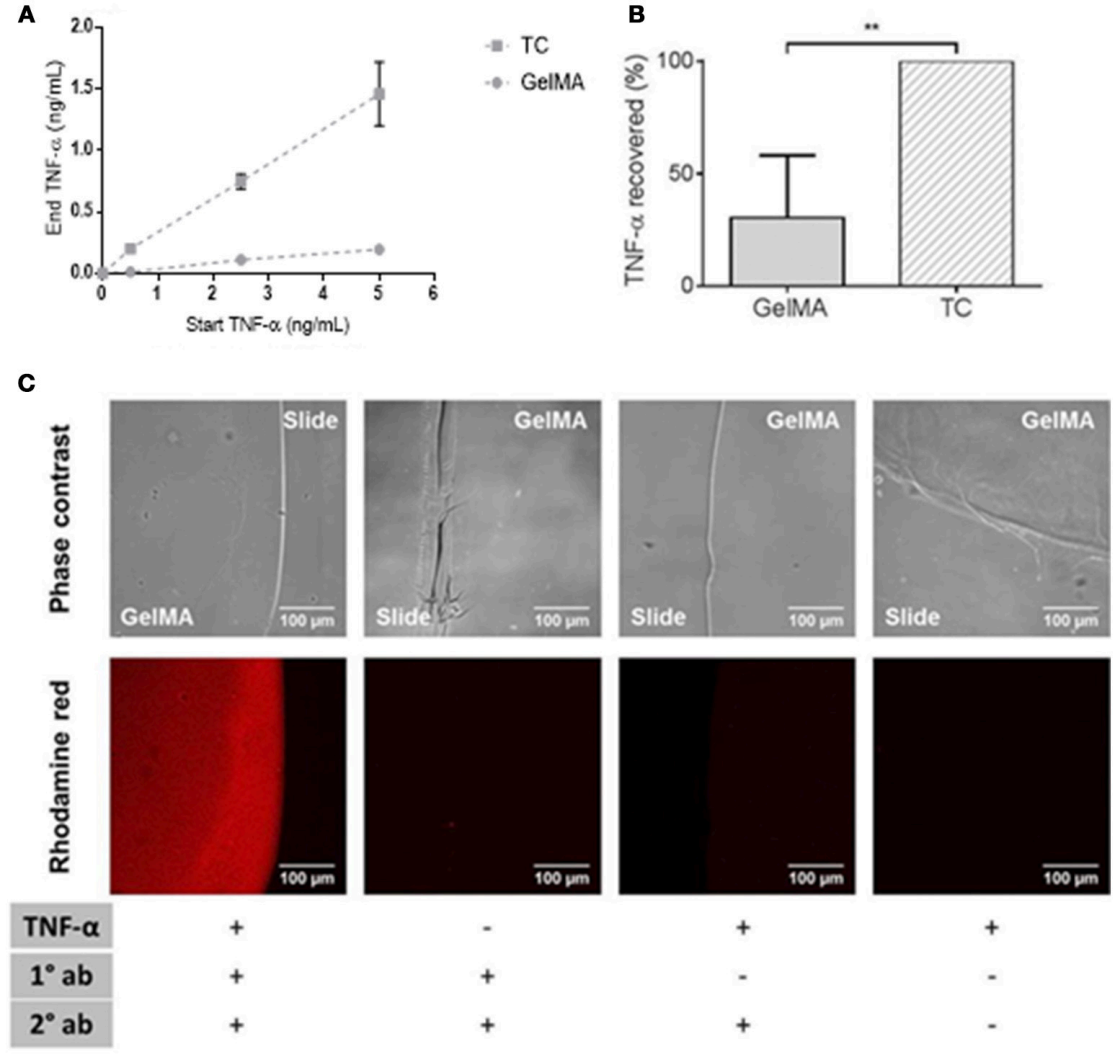
Depletion of soluble TNF-α by GelMA hydrogels. **(A)** Residual TNF-α detected after incubation of GelMA hydrogels in media containing 0.5, 2.5, and 5 ng/mL recombinant TNF-α. Error bars represent standard error of the mean. **(B)** TNF-α recovered from TC (100%) and GelMA (30%), a significant reduction in residual TNF-α recovered from GelMA was found relative to TC (**P = 0.0006). A two-tailed *t*-test was performed to determine statistical significance (*n* = 3 independent experiments). Error bars represent standard deviation. **(C)** TNF-α-treated GelMA, labeled with mouse anti-human TNF-α (primary) and rhodamine red goat anti-mouse (secondary) antibodies. Untreated GelMA, stained with primary and secondary antibodies. GelMA, secondary antibody control. GelMA, unlabelled. Images acquired on an LSM710 META Zeiss confocal system, using a 20X objective. Scale bars represent 100 μm.

As shown in Figure 4, there was a large reduction in soluble TNF-α detected in supernatants collected from GelMA coated wells compared to TC. This was shown over a titration of concentrations of TNF-α in Figure 4A. By calculating the residual TNF-α in the media collected from GelMA (30%), relative to TC (100%), a significant reduction of TNF-α recovered from GelMA was determined (^**^P = 0.0006) as shown in Figure 4B. To confirm the fate of the TNF-α protein, GelMA was incubated with the TNF-α-containing media for 4 h. Immuno-fluorescent staining was performed for TNF-α in order to determine whether it was bound within the gel. As shown in Figure 4C, GelMA hydrogels which had been incubated with TNF-α were positively and specifically stained for human TNF-α, indicating the presence of TNF-α bound within the hydrogel.

## Discussion

A number of factors can determine the cytotoxicity of biomaterials, for example, by-products of degradation. Therefore, cytotoxicity is a critical parameter when assessing the suitability of a biomaterial for *in vivo* and *in vitro* applications (Williams, 2008). In our experiments, quantification of apoptotic and necrotic cells, using Annexin V/PI staining, showed a comparable percentage of dead cells on GelMA and TC plastic after 5 days of culture (Figures 1A–C) indicating that GelMA hydrogels did not have a detrimental impact on the health of immune cells compared to conventional TC. This was further confirmed by direct visualization of live and dead cells *in situ* using a LIVE/DEAD assay, which also showed similar levels of cell viability on GelMA and TC (Figure 1D).

Having established the comparable viability of immune cells in TC and GelMA we assessed their pro-inflammatory cytokine profile after stimulation with LPS. In the TC condition, levels of all cytokines increased significantly in response to LPS. In the presence of GelMA hydrogels however background levels of all inflammatory cytokines were raised. This could be explained by either the immunogenicity of xenogeneic proteins in porcine gelatin (Chan and Leong, 2008), which is used as raw material for synthesizing GelMA, or the presence of low-level endotoxin in GelMA preparations. Nevertheless, cells on GelMA still responded to LPS stimulation with an increase in IL-1β, IL-6, and IL-8 production which was on a par with the concentration of these cytokines from cells cultured on TC. However, levels of TNF-α in the supernatant of cells cultured on GelMA did not change in response to LPS stimulation and was significantly lower than cells cultured on TC (Figure 2). Within the PBMC population, monocytes are the main responders to LPS (Tazi et al., 2006) and are largely accountable for the production of pro-inflammatory cytokines particularly TNF-α. Therefore, we also examined the level of TNF-α production in response to LPS on TC and GelMA using purified monocytes. To rule out the possibility of LPS being adsorbed on GelMA, hence lower levels of TNF production in these experiments, monocytes were first stimulated with LPS (for 1 h) before being transferred to either TC plates or GelMA. Data from these experiments also showed significantly lower levels of TNF-α detected in the supernatant of monocytes cultured on GelMA compared to TC plates. Interestingly, the same pattern was also observed when monocytes were seeded on an enzymatically cross-linked gelatin hydrogel (Figure 1E).

Given the comparable viability of cells cultured on TC and GelMA but significant reduction in soluble TNF-α in GelMA condition, it was reasonable to assume that GelMA could actually mop up TNF. Indeed data presented in Figure 4 clearly show that GelMA seems to act as a sink for TNF-α (Figure 4C) which leads to a significant reduction in soluble TNF-α in the media (Figures 4A,B) decreasing the bio-availability of soluble TNF for interaction with immune cells in suspension. Collagen has previously been demonstrated to have similar modulatory properties over certain pro-inflammatory cytokines with clinically relevant effects (Wiegand et al., 2010). Since GelMA is gelatin based, it seems feasible for GelMA to possess similar cytokine-binding properties. Gelatin has a repeating amino acid sequence of Gly-Pro-X and contains many chemical side groups (Van Den Bulcke et al., 2000). The functional groups are likely to interact non-covalently with a variety of small molecules and form macromolecular complexes with the proteins to which it binds by means of hydrogen links, providing a potential mop up mechanism (Taravel and Domard, 1993; Nezu and Winnik, 2000; Vaidyanathan et al., 2003; Frasca et al., 2012). In addition, the three dimensional (3D), porous nature of the GelMA hydrogel could also assist in the wicking away of cytokines (Benton et al., 2009). However, more detailed studies may be required to elucidate the exact nature of TNF-α interaction with GelMA.

To better understand observed changes in TNF-α production in immune cell cultured on GelMA we also investigated TNF-α mRNA expression using conventional and real-time PCR. Data from these experiments showed an increase in TNF-α mRNA expression within 30 min of LPS stimulation in both TC and GelMA cultures, indicating that TNF-α gene expression was up-regulated. However, interestingly at 4-h time point, the levels of TNF-α mRNA in cells cultured on GelMA hydrogels was reduced to the levels observed in unstimulated cells, suggesting TNF-α gene expression had been suppressed in these cells. In contrast, LPS-stimulated cells cultured in the TC condition maintained high expression of TNF-α (Figure 3), albeit at lower levels compared to 30 min. It is important to highlight that TNF mRNA expression in response to LPS stimulation, and in the absence of other stimulatory signals (e.g., IFNs), peaks at 2 h post-stimulation (Kohn et al., 1992) which explains the observed decrease in mRNA expression in LPS stimulated cells on TC at 4hr time point.

Collectively these data indicate that while GelMA hydrogels induce an inflammatory cytokine profile under resting conditions, they seem to reduce the availability of soluble TNF-α following stimulation with LPS, showing that GelMA has the potential to modulate the production of a key regulator of the immune system under pro-inflammatory conditions. TNF-α has diverse roles in the function and regulation of the immune system, dependent on tissue type, immunological context and timing (Wajant et al., 2003). It predominantly has pro-inflammatory effects in affected tissues and can exacerbate, or lead to the progression of disease, for example, rheumatoid arthritis and chronic wounds (Albillos et al., 2004).

**Figure 5:**
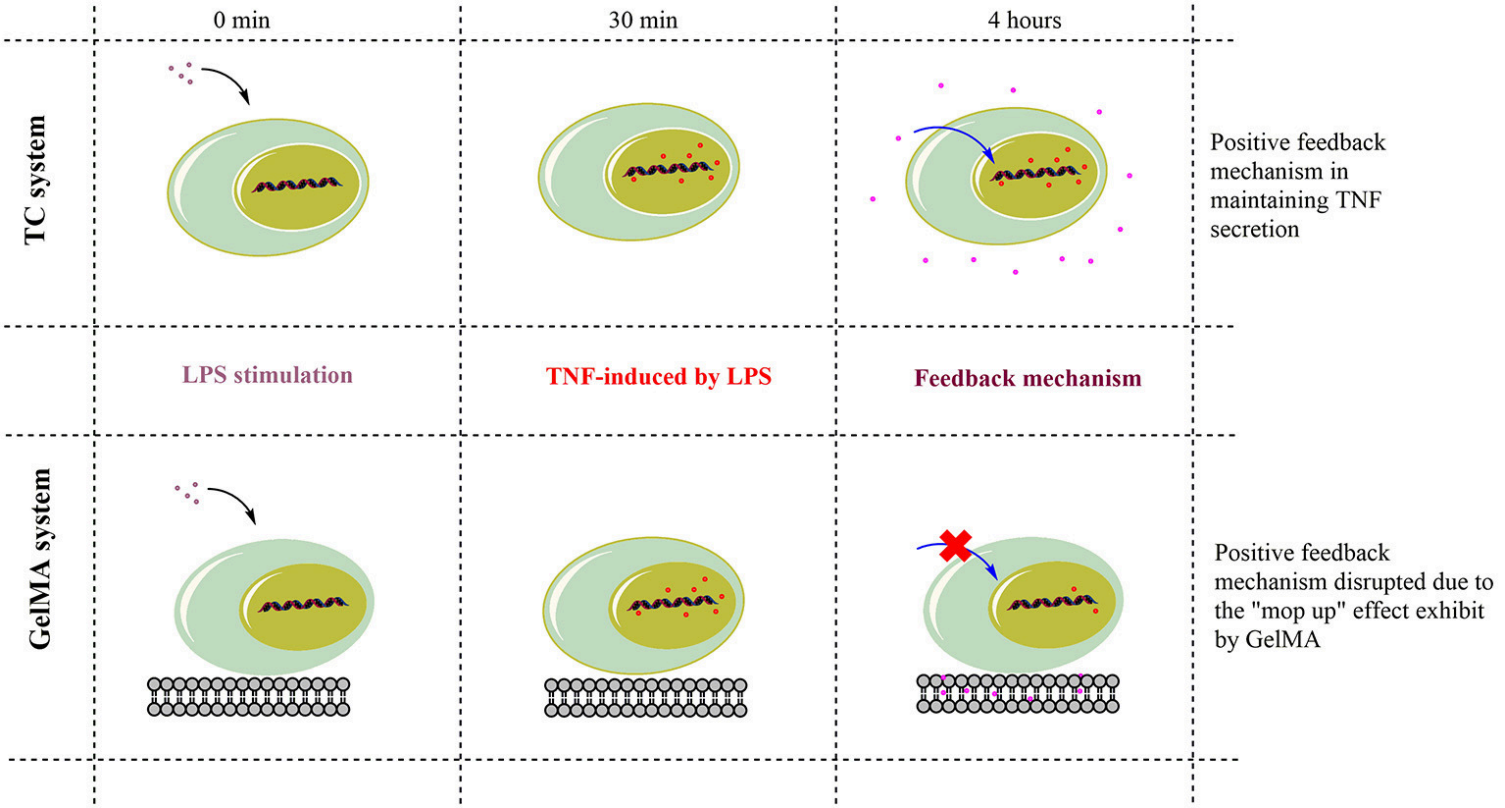
Schematic action mechanism for GelMA in the modulation of TNF-α. TNF-α gene expression increased within 30 min of LPS stimulation in both TC and GelMA conditions. After 4 h, LPS-induced TNF-α expression is maintained through a positive feedback mechanism and levels of secreted TNF-α peak in TC. Whereas the GelMA hydrogel sequesters secreted TNF-α protein, thereby effectively removing it from solution. This disrupts the relay of positive feedback signals to the cell for the maintenance of TNF-α expression. Thus, by 4 h, in cells cultured on GelMA TNF-α mRNA levels are suppressed to the levels of un-stimulated cells and there is also no detectable increase in soluble TNF-α in the supernatant.

Therefore, TNF-α is under strict regulatory mechanisms which control its expression. For instance, the signaling pathways involved in TNF-α production include MAP kinases (ERK1/2, p38 and JNK) and NF-kappaB (Tazi et al., 2006). Soluble TNF-α signals through the receptor TNF-R1, which in turn activates a variety of signal transduction pathways, including MAP kinases and NF-kappaB. These in turn regulate transcription factors involved in the production of inflammatory cytokines, such as TNF-α (MacEwan, 2002; Ronkina et al., 2010). Indeed, different studies have shown that soluble TNF-α provides positive feedback to cells via paracrine or autocrine signaling in order to maintain expression during acute inflammation (Wu et al., 1993; Coward et al., 2002; Guergnon et al., 2003; Yarilina et al., 2008; Gane et al., 2016). Thus, while understanding the full mechanism of TNF-α suppression on GelMA may need further investigations, we propose that TNF-α sequestration in GelMA reduces the availability of soluble TNF-α hence switching off such a feedback mechanism resulting in the suppression of TNF-α gene expression and potential dampening of TNF-α production (Figure 5). Cross-linked gelatin is more resistant to degradation by proteolytic enzymes such as gelatinase and collagenases. For example enzymatic degradation of GelMA hydrogels with collagenase II showed ~30% mass loss during 14 days incubation period (Kang et al., 2014). Therefore, due to its low degradation rate, GelMA can sequester TNF-α before potential release of TNF-α due to GelMA degradation. This together with the short bioavailability and TNF-α half-life (5–10 min; Kamada et al., 2000; Harrison et al., 2004) means it is unlikely that sequestered TNF-α will be released back to the culture media and/or have any biological activity. However, the potential activity of the sequestered TNF-α in GelMA remains unknown and further studies are needed to fully understand this process.

This potentially anti-inflammatory property of GelMA could be harnessed. For example, in situations where excess TNF-α has a role in disease, such as severe tissue damage, the ability of GelMA to both mop up TNF-α and subsequently suppress expression in a localized environment could be beneficial. Previous work have shown that biomaterials can have profound influences on immune cell behavior, such effects must be taken into consideration when choosing biomaterials for applications where the immunological output is key to the functionality of the construct (Singh and Peppas, 2014). We anticipate research in this area will have significant implications, particularly since *in vivo* applications are becoming closer to reality with the development of increasingly complex biologically engineered constructs. This study contributes to a recent trend in the characterization of biomaterials for culture with immune cells. Although work in this area is still in the early stages, studies such as this highlight the importance of fully understanding the immunogenic properties of biomaterials to ensure the scaffold is appropriate for the required application.

## Ethics statement

This study was carried out in accordance with the recommendations of Faculty of Medicine and Health Services Research Ethics Committee. The protocol was approved by the Faculty of Medicine and Health Services Research Ethics Committee, University of Nottingham. All subjects gave written informed consent in accordance with the Declaration of Helsinki.

## Author contributions

ARD, PVB, CET, DA, and LH performed experiments and analyzed data. MN and AK provided reagents and contributed to data analyses. AMG, FR, and CA designed the study and contributed to data analyses. ARD, CET, and AMG wrote the manuscript. All authors read and approved the manuscript.

### Conflict of interest statement

The authors declare that the research was conducted in the absence of any commercial or financial relationships that could be construed as a potential conflict of interest.
